# GQ style: a multipronged therapeutic approach to pulmonary arterial hypertension

**DOI:** 10.1007/s00424-024-03056-2

**Published:** 2024-12-17

**Authors:** Wolfgang M. Kuebler

**Affiliations:** Institute of Physiology, Charité-Universitätsmedizin Berlin, Berlin, Germany

Pulmonary arterial hypertension (PAH) comprises a group of diseases characterized by an elevated mean pulmonary artery pressure > 20 mmHg, a pulmonary vascular resistance > 2 Wood units, and a pulmonary arterial wedge pressure ≤ 15 mmHg [1]. PAH results from a combination of increased vascular tone and extensive vascular remodeling in pulmonary resistance vessels with medial and intimal hypertrophy of arteri(ol)es, muscularization of distal lung arterioles, loss of functional (micro-)vessels, and reduced arterial compliance [1]. Until the recent advent of activin signaling inhibitors as a new class of PAH drugs, all clinically approved therapies have primarily focused on counteracting the imbalance of vasoconstrictive versus vasodilatory mediators in PAH, and as such, have been able to alleviate symptoms and to decelerate the disease course, yet not to revert it [1].

In a comprehensive study published in *EMBO Molecular Medicine*, Seidinger and colleagues now provide proof-of-principle for a new therapeutic strategy targeting Gαq protein-mediated G-protein coupled receptor (GPCR) signaling [2]. Gαq-coupled receptors are critically implicated in the pathogenesis of PAH as key agonists of pulmonary vasoconstriction and vascular remodeling including serotonin, angiotensin II, endothelin-1, thromboxane, thrombin, or sphingosine-1-phosphate act via Gαq signaling, and since PAH is frequently associated with the emergence of agonistic antibodies directed against Gαq-coupled receptors including endothelin-1 receptor type A, angiotensin II receptor type 1, or sphingosine-1-phosphate receptors type 2 and 3 [3, 4]. As such, Gαq inhibition may present a novel promising approach for the treatment of PAH.

Based on this rationale, Seidinger and colleagues tested the effects of the depsipeptide FR900359, a pan-Gαq inhibitor which prevents GDP release from Gαq and thus freezes the Gαβγ heterotrimer in an inactive confirmation [5]. In wire myograph assays, FR900359 reversed or prevented, respectively, vasoconstriction of murine and porcine pulmonary arteries in response to various PAH-associated Gαq-coupled GPCR agonists including serotonin, endothelin-1, or the thromboxane analog U46619 with the latter two responses also being partially mediated by Gα12/13 signaling. These effects could be replicated in murine and human precision-cut lung slices and in isolated-perfused mouse lungs. In preconstricted murine pulmonary arteries, FR900359 was even more effective as a vasodilator than a triple combination of bosentan, iloprost, and sildenafil, the presently most effective clinical vasodilator therapies in PAH.

Moving on to an in vivo model of pulmonary hypertension, the authors show that acute Gαq inhibition by either intratracheal or intraperitoneal application of FR900359 reduced right ventricular systolic pressure in chronic hypoxic mice. In addition to these acute vasodilatory effects, FR900359 also effectively targeted lung vascular remodeling: In a murine model of pulmonary hypertension induced by the VEGF-receptor antagonist SU5416 in combination with hypoxia (Su/Hx), chronic Gαq inhibition by daily intraperitoneal injection of FR900359 prevented at large the characteristic hallmarks of pulmonary hypertension, i.e. the increase in right ventricular systolic pressure, right ventricular cardiomyocyte hypertrophy, and medial hypertrophy of pulmonary arteries and arterioles. At the cellular level, these effects could be linked to an attenuating effect of FR900359 on pulmonary artery smooth muscle proliferation and migration in response to Gαq-coupled GPCR agonists such as serotonin in vitro. Of particular translational relevance, FR900359 also partially reversed pulmonary hypertension in Su/Hx mice when administered at a time point when pulmonary hypertension was already hemodynamically manifest.

As such, Gαq inhibition presents a promising novel, multipronged therapeutic approach for the treatment of PAH that may be superior to conventional therapies in that it targets several GPCR-mediated mechanisms of pulmonary vasoconstriction and vascular remodeling in parallel. The effectiveness of this approach may extend beyond PAH to other forms of pulmonary hypertension such as pulmonary hypertension with lung diseases and/or hypoxia (group 3 pulmonary hypertension) and other chronic lung diseases as lung airway remodeling is also mediated in part via Gαq [6]. Clinical applicability of this approach depends, however, critically on the potential adverse effects of pan-Gαq inhibition in the lungs, the blood, and the systemic circulation. For the pulmonary circulation, Seidinger and colleagues report that FR900359 did not inhibit hypoxic pulmonary vasoconstriction (HPV) in isolated-perfused murine lungs, suggesting that Gαq inhibition may not interfere with physiological ventilation-perfusion matching. This finding is unexpected as HPV has previously been linked to activation of phospholipase C [7, 8], and may implicate the involvement of PLC isoforms other than PLC-β (the direct downstream target of Gαq) in HPV. In the systemic circulation, both acute and chronic intraperitoneal administration of FR900359 reduced left ventricular systolic pressure to levels of approximately 75 mmHg highlighting systemic hypotension as an expected adverse effect. Systemic administration of Gαq inhibitors may also attenuate GPCR agonist responses in circulating blood cells, such as platelet activation and aggregation or monocyte responses including cytokine production, chemotaxis, and phagocytosis. In line with this view, FR900350 treatment attenuated the infiltration of monocytes/macrophages into the lungs of Su/Hx mice. While it may be argued in view of the emerging role of immune cells in the pathophysiology of pulmonary hypertension [9, 10] that some of these side effects may be beneficial, lung specific targeting of Gαq inhibitors seems a safer and, as demonstrated by Seidinger and colleagues, equally effective approach. As such, one can hope that this elegant work will stimulate the development of formulations that allow for lung-targeted administration of Gαq inhibitors e.g. by aerosolization.

## Data Availability

No datasets were generated or analysed during the current study.
